# Risk of magnetic resonance imaging-induced magnet dislocation for different types of cochlear implants: a single-center retrospective study

**DOI:** 10.1186/s40463-023-00633-w

**Published:** 2023-04-21

**Authors:** Robin Rupp, Matthias Balk, Matti Sievert, Victoria Leibl, Stephan Schleder, Moritz Allner, Heinrich Iro, Ulrich Hoppe, Joachim Hornung, Antoniu-Oreste Gostian

**Affiliations:** 1 Department of Otorhinolaryngology, Head and Neck Surgery, Friedrich-Alexander-University Erlangen-Nürnberg (FAU), Waldstraße 1, 91054 Erlangen, Germany; 2 Department of Diagnostic and Interventional Radiology, Merciful Brothers Hospital St. Elisabeth, 94315 Straubing, Germany

**Keywords:** Cochlear implant, Magnetic resonance imaging, Complication, Magnet dislocation, Magnet repositioning, CI24RE, CI500, Synchrony

## Abstract

**Background:**

When performing magnetic resonance imaging (MRI) in patients with a cochlear implant (CI), complication rates vary widely in the literature. The primary objective of this retrospective study was to determine the prevalence of complications, in particular magnet dislocation, in patients with CI undergoing 1.5 Tesla (T) MRI. As a secondary objective, the prevalence of magnet dislocation for specific cochlear implant device types was elaborated.

**Methods:**

In a single-center retrospective study, all patients with a cochlear implant presenting for an MRI examination at 1.5 T at our institution between January 1st, 2010 and December 31st, 2020 were included. Implants with axial and diametrical magnets were included in the study. MRI safety measures were applied before imaging. The prevalence of complications was evaluated. Magnet dislocation rates were calculated for device types with at least 20 MRI exposures.

**Results:**

During the study period, 196 MRI examinations were performed in a total of 128 patients, accounting for 149 different implants (21 implanted bilaterally) with a total of 231 implant exposures to MRI (average 1.69 ± 1.57; min. 1, max. 12). Complications were reported in 50 out of 231 cochlear implant exposures (21.6%). Magnet dislocation occurred in a total of 27 cases (11.7%). Dislocation rates were 29.6% for the Cochlear® CI500 series (24 dislocations from 81 exposures), 1.1% for the Cochlear® CI24RE series (1 from 87) and 0% for the MED-EL® Synchrony (0 from 36). The dislocation rate for the CI500 was significantly higher than for the CI24RE (χ^2^_(1)_ = 26.86; *p* < 0.001; ϕ = 0.40) or the Synchrony (χ^2^_(1)_ = 13.42; *p* < 0.001; ϕ = 0.34).

**Conclusions:**

For 1.5 T MRI, the risk of magnet dislocation ranges from 0 to 29.6% and depends on the CI device type. Implants with a diametrical magnet can be considered potentially MRI-safe, whereas in CIs with axial magnets, the CI500 is at high risk of magnet dislocation. Therefore, apart from a strict indication for an MRI and adherence to safety protocols, post-MRI follow-up examination to rule out magnet dislocation is recommended.

**Graphical Abstract:**

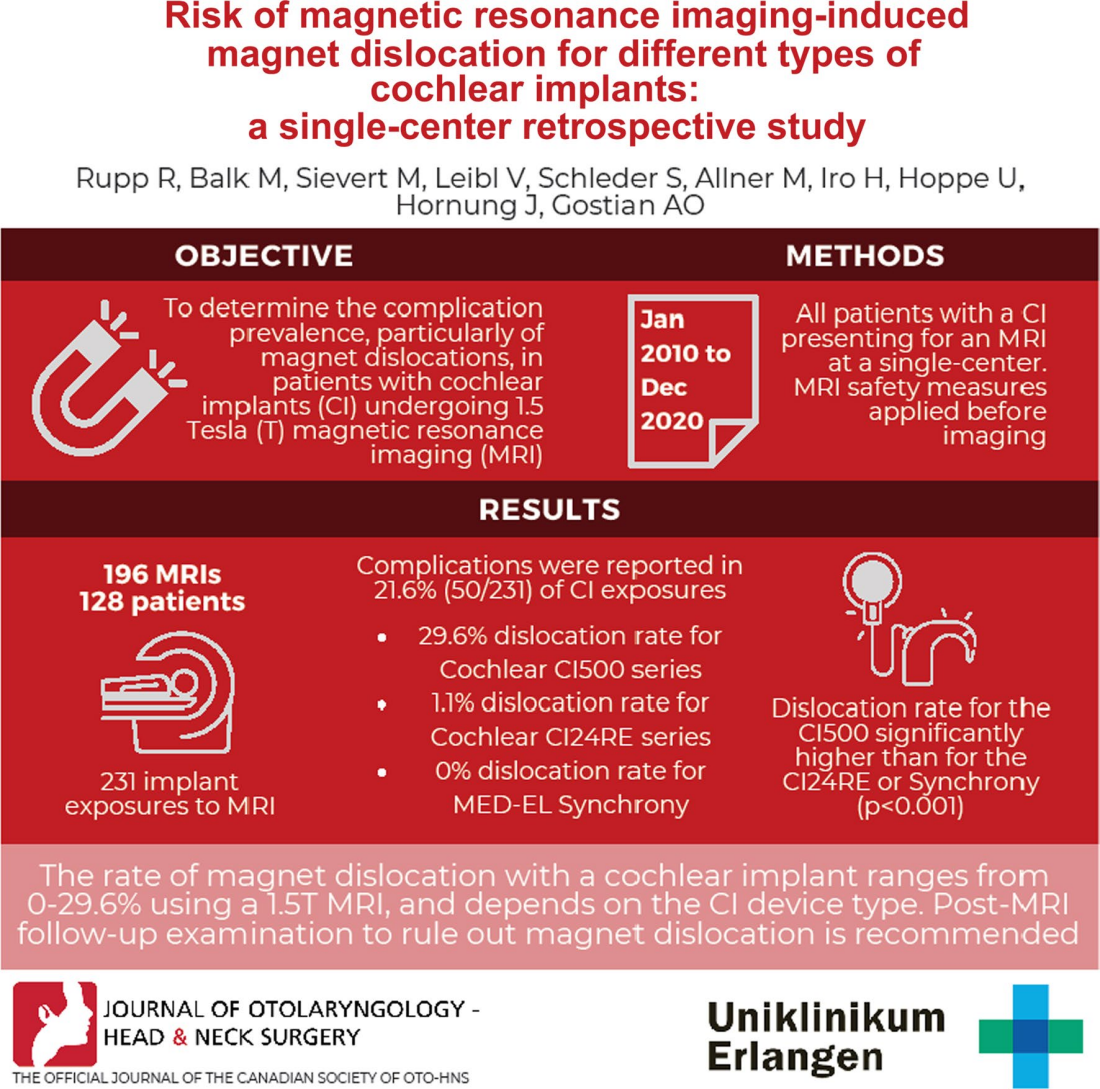

## Introduction

Early cochlear implant (CI) types contained an internal magnet that was fixed to the housing and could not be removed. The development of surgically removable axial magnets was one step in the evolution of magnetic resonance imaging (MRI)-conditional implants with the possibility of reducing the image artefact surrounding the device by removing the magnet [1]. In general, the application of a compression bandage is recommended during MRI examination at 1.5 Tesla (T) to prevent complications caused by the magnetic field [2, 3]. Nevertheless, dislocation of the internal magnet caused by MRI has been described for different implant types from two major CI manufacturers. For devices from Cochlear® (Cochlear Limited, Sydney, Australia), dislocations have been reported for implants from the Nucleus® Series (CI24M/CI422, CI24R, CI24RE) and Nucleus® Profile™ Series (CI500) as well as for the auditory brain stem implant ABI24M [4–9]. For implants from Advanced Bionics® (Advanced Bionics, Valencia, United States), dislocations are described for the HiRes 90 K, HiRes 90 K Advantage and HiRes Ultra [4, 5, 9–11]. Dislocation of the magnet usually requires immediate treatment, as prolonged dislocation may lead to magnet extrusion causing implant infection with subsequent loss of the device [8]. Surgical magnet repositioning is performed frequently but bears the risk of subsequent implant infection and the need for device explantation [5]. Manual repositioning by applying pressure from the outside is an option in magnets that are only partial dislocated [12–14]. In 2014, MED-EL® (MED-EL, Innsbruck, Austria) introduced Synchrony, a CI with a new type of removable internal magnet with a diametric polarization that is freely rotatable and aligns itself to the magnetic field. A compression bandage is not mandatory for MRI examinations up to 3 T [15]. To date, magnet dislocation has not been reported for this type of magnet [10, 13, 16, 17]. Other manufacturers followed this shift toward a safer MRI implant: the Nucleus® Profile™ Plus Series by Cochlear® with a diametric, self-aligning magnet with no need for a compression bandage acquired FDA approval up to 3 T in 2019 [18]. Advanced Bionics® introduced the HiRes Ultra 3D with four cylindrical, freely rotatable diametric magnets; it is MRI-compatible without a compression bandage up to 3 T [3, 19]. Nevertheless, many devices implanted over the last one and a half decades still contain axial magnets bearing the risk of magnet-related complications like demagnetization, polarity reversal, and dislocation. In this context, the risk of magnet dislocation is reported to be approximately 6.5% according to 10 different cohort studies [1], but varies from 0 to 15% among these studies. In some studies, no detailed information on the affected CI device types is given [4, 14, 20, 21]. Due to these inhomogeneities concerning the reported risk of magnet dislocation in the literature, a closer examination of dislocation rates for specific implant types is necessary and clinically important. Therefore, the objective of this retrospective study was to determine the prevalence of complications when performing MRI in patients with a CI and to elaborate the prevalence of magnet dislocation for specific types of CI devices.

## Materials and methods

### Patients and examinations

This retrospective study was conducted at a tertiary referral medical center (Department of Otorhinolaryngology and Head and Neck Surgery, Friedrich-Alexander-Universität Erlangen-Nürnberg (FAU), Erlangen, Germany) and was approved by the institutional review board (application number: 99_21 Bc).

All patients with a CI presenting for an MRI examination at our institution between January 1st, 2010 and December 31st, 2020 were eligible for the study. Inclusion criteria were as follows: CI present; at least one MRI performed at 1.5 T at our institution with internal magnet in place; application of compression bandage if necessary; complete medical record. The following exclusion criteria applied: MRI not performed at our institution; removal of internal magnet prior to MRI; incomplete medical record.

Prior to MRI examination, the device type was identified before checking whether the implant was MRI-conditional according to the current recommendations of the manufacturer. For application of the compression bandage, the exact location of the internal magnet was identified using the magnet of the outer processor. Generally, the compression bandage was applied with the Cochlear™ Nucleus® Implant Bandage and Splint Kit for MRI (Cochlear Ltd., Sydney, Australia) with an elastic bandage and a counter-pressure element in the shape of a credit card. The updated MRI kit has been available in Europe since 2020 and now contains a cylindrical counter-pressure element with a magnet for easy localization of the internal implant magnet [2]; the downside of this additional magnet is an enlarged artefact, so that it was not used when performing head MRI. Furthermore, a piece of A4 paper was used that was folded 5 times and fixed over the magnet as a counter-pressure element with a cohesive bandage.

In case of pain, swelling, vertigo or reduced CI performance after MRI, diagnostic workup included a thorough examination by an ear, nose and throat specialist of the department. In general, ultrasound examination was performed in the case of suspected magnet dislocation. If magnet dislocation was confirmed, a surgical procedure or a manual repositioning maneuvre was performed.

The number of performed MRI scans was determined for every single device included in the study. The total number of CI devices exposed to MRI was then calculated for each specific CI type. Magnet dislocation rates were calculated for CI types with ≥ 20 MRI exposures.

Body regions of MRI examinations were grouped as follows: head; cervical/thoracic spine; lumbar spine/pelvis/abdomen; arm; leg. The dislocation rate of the head region was compared to that of the rest of the body. Additionally, the dislocation rate of the head and trunk region (cervical/thoracic spine and lumbar spine/pelvis/abdomen) was compared to that of the extremities.

### Statistical analysis

Metric variables are presented as mean values ± 1 standard deviation (SD), minimum (min.) and maximum (max.). Categorical variables were reported as absolute frequencies (n) with percentages (%). Statistical calculations were performed using SPSS (IBM SPSS Statistics 22.0, IBM, New York, NY). The Chi-square test was used for the comparison of nominal variables. If the reported frequency of magnet dislocation was below 5, Fisher’s exact test was conducted. A *p*-value ≤ 0.05 was considered as statistically significant. For nominal variables, the effect size ϕ was calculated at a post-hoc level, with ϕ = 0.1 displaying a small effect, ϕ = 0.3 representing a medium and ϕ = 0.5 a strong effect.

## Results

### Patient and implant characteristics

**Fig. 1:**
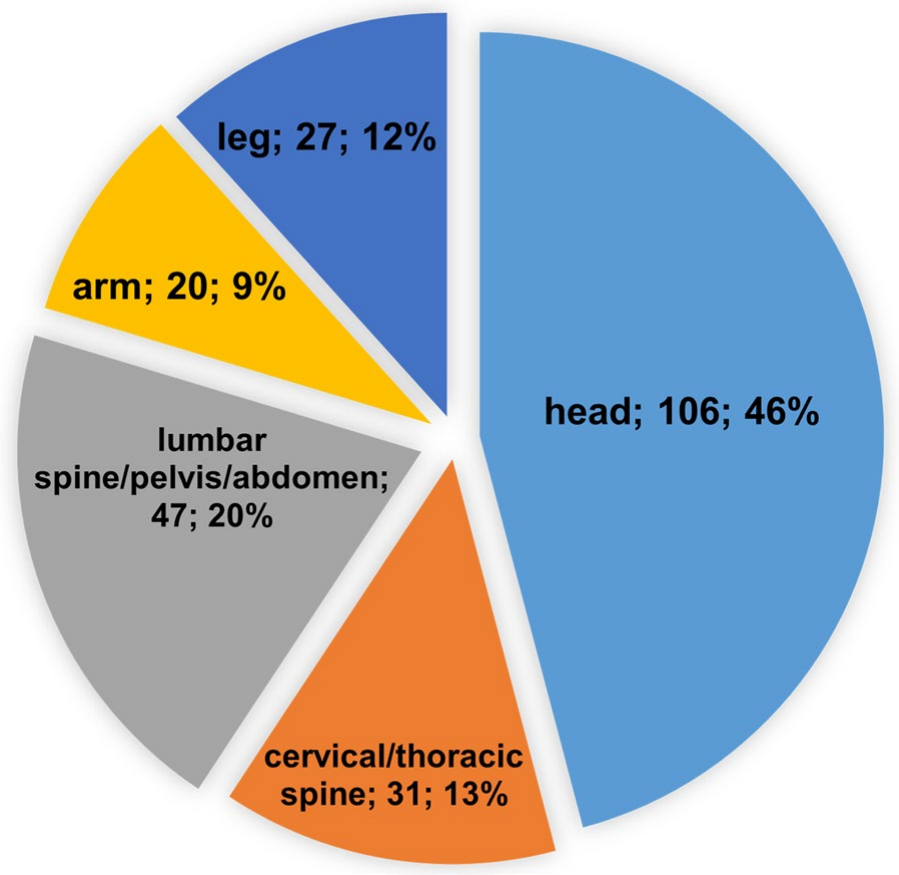
Body regions of all 231 CI exposures; body region scanned, number of MRI exposures, percentage of all MRI exposures; CI, cochlear implant; MRI, magnetic resonance imaging

During the study period, 196 MRI examinations were performed in a total of 128 patients, accounting for 149 different implants (78 right-sided implants). The 128 patients included (54 ♀; 42%) averaged 57.0 ± 19.1 years (yr.) (min. 3.2 yr., max. 92.5 yr.) at the time of the first MRI examination. The mean time from first implantation to first MRI examination was 4.2 ± 3.3 yr. (min. 1.7 months, max. 16.6 yr.). Considering that 21 patients had bilateral implants and 42 patients underwent more than one MRI examination (average 1.69 ± 1.57; min. 1, max. 12), there were a total of 231 implant exposures to MRI. Of the 231 CI exposures to MRI, the anatomical regions scanned were as follows: 106 scans of the head (46%), 31 scans of the cervical and thoracic spine (13%), 47 scans of the lumbar spine, pelvis and abdomen (20%), 20 scans of the arm (9%) and 27 scans of the leg (12%). Figure 1 shows the distribution of all 231 CI exposures on the different body regions that were scanned.

Device exposure to MRI (n = 231) was as follows: 174 exposures (75.3%) with implants from Cochlear® (CI24RE = 87 (37.7%); CI500 = 81 (35.1%); CI24M = 4 (1.7%); CI24R = 1 (0.4%); CI600 = 1 (0.4%)), 49 exposures (21.2%) with implants from MED-EL® (Synchrony = 36 (15.6%); Concerto = 11 (4.8%); Sonata = 2 (0.9%)), and 8 exposures (3.5%) with implants from Advanced Bionics® (HiRes 90 K = 6 (2.6%); HiRes Ultra = 2 (0.9%)).

**Fig. 2:**
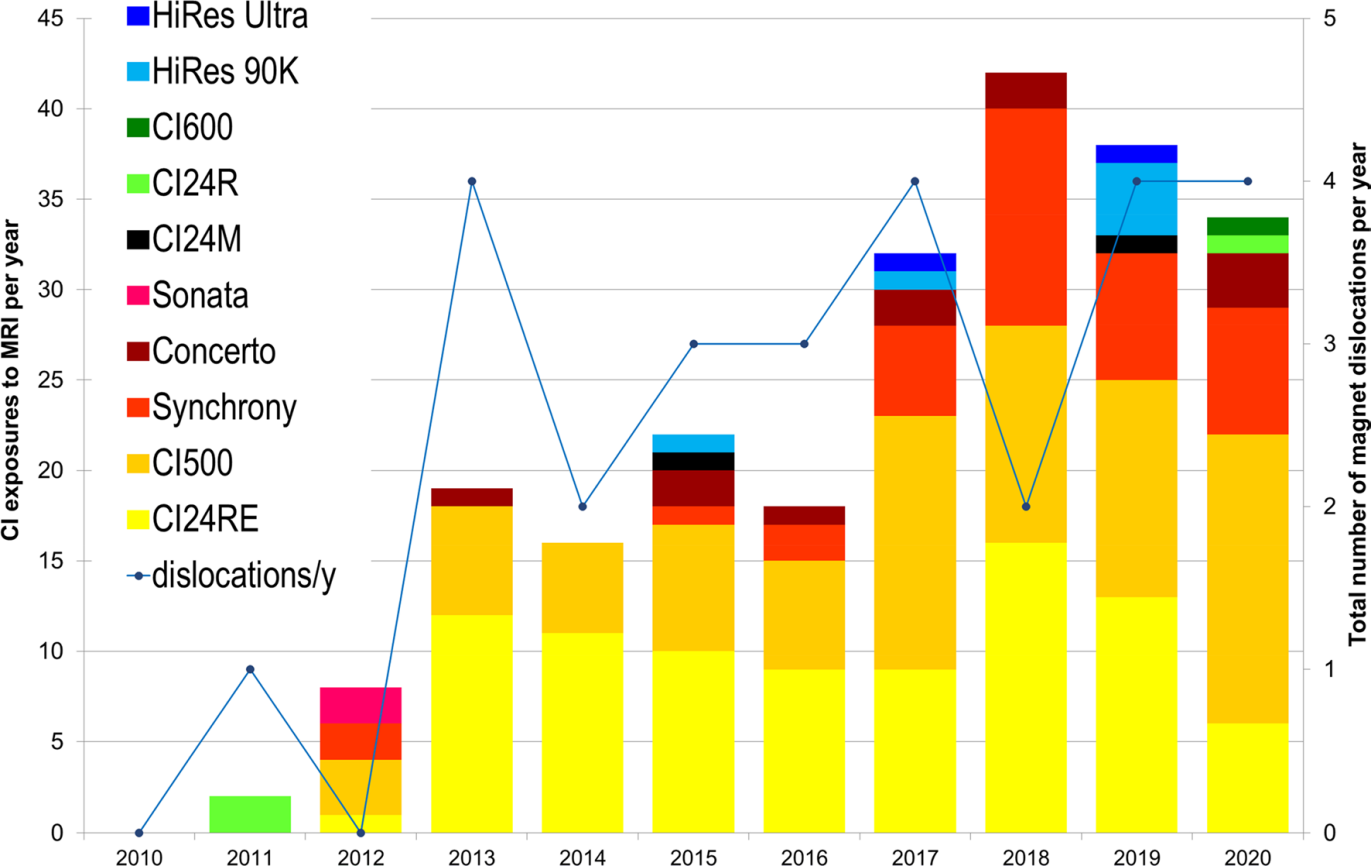
CI exposures to MRI per implant type per year and total number of magnet dislocations per year; x-axis: year of MRI; left y-axis: number of CI exposures to MRI per year; right y-axis: total number of magnet dislocations per year; CI, cochlear implant; MRI, magnetic resonance imaging; y, year

Figure 2 shows CI exposures for the different implant types and the total number of magnet dislocations per year.

### Complications

Complications after MRI were reported in 50 out of 231 CI exposures (21.6%) and included pain and/or swelling at the implant area (n = 14/ 6.1%), vertigo (n = 3/ 1.3%), dysesthesia of the skin covering the implant (n = 1/ 0.4%), termination of MRI examination ahead of time because of pain (n = 4/ 1.7%) and magnet dislocation (n = 27/ 11.7%). No MRI-induced magnet weakening or polarity reversal was reported. The 27 cases of magnet dislocation occurred in 20 different implants, accounting for 20 patients, and affected implants with axial magnets only. In 18 implants the magnet dislocated once, whereas in another implant the magnet dislocated twice, and in one additional implant seven times during various MRI examinations. One dislocation each occurred in CI24M, CI24R and CI24RE devices; all other dislocations (n = 24) appeared in 17 different CI500 implants. Dislocation rates for implant types with ≥ 20 MRI exposures were 29.6% for the CI500 (24 dislocations out of 81 exposures), 1.1% for the CI24RE (1 out of 87) and 0% for the Synchrony (0 out of 36).

The dislocation rate for the CI500 was significantly higher than for the CI24RE (χ^2^_(1)_ = 26.86; *p* < 0.001; ϕ = 0.40) or the Synchrony (χ^2^_(1)_ = 13.42; *p* < 0.001; ϕ = 0.34) as displayed by medium effect sizes. No difference in the dislocation rate was found between CI24RE and Synchrony (Fisher’s Z: *p* = 0.707; ϕ = 0.06), resulting in a small effect size.

The dislocation rate of the head region (106 exposures with 15 dislocations; 14.2%) compared to the rest of the body (125 exposures with 12 dislocations; 9.6%) showed no significant difference (χ^2^_(1)_ = 1.15; *p* = 0.283; ϕ = 0.07). However, when comparing the head and trunk region (147 exposures with 26 dislocations; 17.7%) with the extremities (47 exposures with one dislocation; 2.1%), significant more dislocations occurred (χ^2^_(1)_ = 5.23; *p* = 0.022; ϕ = 0.15).

Magnets were repositioned successfully in all 27 cases of dislocation. In 23 out of 27 cases, surgical repositioning was performed under local anesthesia. The remaining 4 magnets were treated effectively in a manual repositioning maneuvre; success was controlled via transcutaneous ultrasound. The functionality of all 27 implants could be preserved completely by treating the dislocations.

**Fig. 3:**
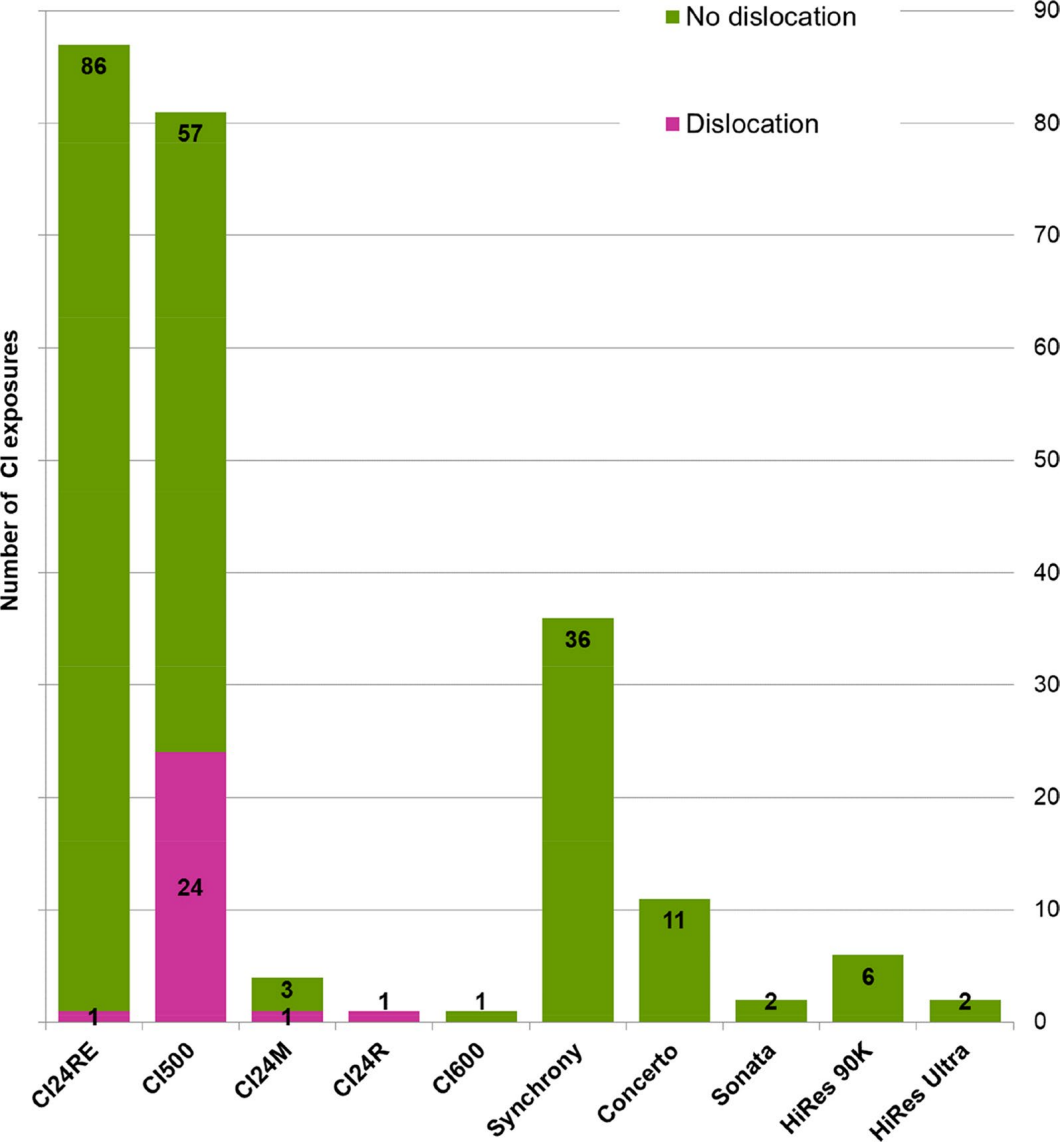
Number of CI exposures to MRI by implant type including the number of dislocations recorded; CI, cochlear implant; MRI, magnetic resonance imaging

Figure 3 presents the number of CI exposures to MRI by implant type including the number of dislocations recorded.

## Discussion

The present study investigated complications, in particular the risk of magnet dislocation in patients with CI who underwent MRI for any reason in a single tertiary referral center. The overall complication rate was found to be 21.6%. Magnet dislocation occurred in 11.7% of all 231 cases of CI exposure to MRI. All dislocations occurred in implants with axial magnets. The data demonstrate that magnet dislocation is significantly more frequent in CI500 (29.6%) compared with CI24RE (1.1%) and with Synchrony (0%). All 27 dislocated magnets were repositioned successfully while their functionality was maintained.

Complication rates of patients with CI undergoing MRI with magnet in place were reported to vary between 11.7 and 30.8% [6, 10, 13, 22]. This is in line with our result of 21.6%. Loth et al. report in a retrospective questionnaire study that only 11% of MRI examinations could be carried out without any side effects in a group of 91 patients, with pain being the most frequent complication (37%). Grupe et al. even report that 70% of patients suffered from pain during MRI in a series of 33 MRI examinations. Both studies were conducted using a retrospective questionnaire in which patients were actively asked about symptoms, which could be the reason that higher numbers of patients complained of pain.

Dislocation of the internal magnet represents a severe complication in patients with a CI. In fact, it has been described for various implant types with axial magnets [4–11]. In contrast, no dislocations have been described to date for implants with a diametric free-rotational magnet, e.g. MED-EL® Synchrony [10, 16, 17, 23]. In 2015, Carlson et al. examined 34 MRI scans and reported 5 magnet dislocations (14.7%) [6]. An update of this series was given in 2020 by Fussel et al., in which a decreased dislocation rate of 8.9% was observed (14 dislocations in 157 MRI scans) that is comparable to the findings of our study with a dislocation rate of 11.2%. Fussel et al. investigated mostly CI from Cochlear®, but failed to mention the specific device types. As a consequence, they emphasized the need for a subgroup analysis to specify whether the device type or other factors are associated with higher complication rates [13]. Shew et al. reported three magnet dislocations (two in an Advanced Bionics® HiRes Ultra and one in a Cochlear® CI24RE) following 24 MRI examinations, resulting in a dislocation rate of 12.5% [10]. In the study by Grupe et al., three dislocations were seen in 33 scans (9.1%) with no information about the device types [14]. In 2021, Loth et al. reported 111 MRI scans with seven dislocations (6.1%), with more than half of the scans performed with CI from MED-EL®. Interestingly, no dislocation was seen in 20 scans of the CI24RE (0%), whereas seven (36.8%) dislocations occurred in 19 scans of the CI500, which is in line with our results of 1.1% and 29.7%, respectively [24]. In a series of 400 MRI scans on CI and ABI, Tam et al. reported only five dislocations (1.3%). Though, despite of the large number of scans performed, less patients were included compared to the present study (97 vs. 128) and the percentage of scans with the CI500 was only about 7% [4].

In 2020, Young et al. presented a series of seven patients and 17 MRI scans with the MED-EL® Synchrony with no magnet dislocation [17]. Other studies support these findings on the MRI safety of this implant with a diametric, rotatable magnet at 1.5 and 3 T [10, 13, 16, 24–26] and are in accordance with the results of the present study. First clinical and experimental studies on the Advanced Bionics® HiRes Ultra 3D with four cylindrical, rotatable diametric magnets indicate MRI safety without a compression bandage [19, 27, 28].

Apart from clinical data, several experimental studies were recently published focusing on magnet dislocation. In the study by Eerkens et al., the main cause of magnet dislocation was found to be the rotational force induced by the torque experienced inside the magnet bore, which resulted in a possibility of magnet dislocation in implants with axial, removable magnets (CI24RE and HiRes Ultra) and a safe MRI procedure with rotatable magnets (Synchrony and HiRes Ultra 3D) [28]. This is an explanation for higher dislocation rates in MRI of the head and trunk compared to MRI of the extremities where the implant is further away from the magnet bore.

Summarizing these findings, it becomes evident that reported CI magnet dislocation rates greatly depend on the CI device types that are included in a specific series. This, of course, greatly depends on the device types that were implanted at a certain department over a certain period of time. Therefore, in studies with a focus on the complications of MRI, specific CI device types have to be explicitly mentioned to ensure the possibility of comparison between different studies. In this context, this is the first study to elaborate the distinct differences in dislocation rates of the CI500, CI24RE and Synchrony. The results show that magnet dislocation is more prevalent in CI500 than in CI24RE and Synchrony. Therefore, not all implants with axial magnets bear the same risk of dislocation. At this point, it is worth mentioning that the axial magnet of CI500 is slightly thinner and wider than that of CI24RE, which could be a reason for the higher dislocation rate. Additionally, differences in the surrounding silicone housing could influence the probability of magnet dislocation.

In early 2020, Cochlear America changed its MRI safety guidelines for several older implants, including the CI24M, CI24RE and CI500, now recommending magnet removal prior to MRI examination for the US [29]. Following this advice, patients require additional surgery before and after the MRI examination, with the potential risk of implant infection and loss [4]. In children, magnet removal and reinsertion can only be done in general anesthesia. Additionally, tearing of the silastic magnet housing can occur when the magnet is removed [21]. Finally, local wound healing requires a minimum of one week until the CI can be used again. For other regions including Canada and Europe, the MRI Splint Kit is still recommended in the safety guidelines [2, 30]. In this context, the decision on magnet removal prior to MRI should be made individually for every patient.

Regarding these developments, it is recommended to adopt MRI safety protocols that were proposed by several authors [6, 10]. Meticulous counseling of the patient is paramount when planning MRI and, depending on the device type, several questions have to be discussed: (1) Is there a less risky imaging method with the same accuracy in diagnosing the underlying pathology?; (2) Can the magnet remain in situ with the consequence of a larger artefact around the implant or has it to be removed with the risk of implant infection?; (3) How can a post-MRI check-up be ensured to deal with potential complications? We recommend a clinical check-up at the ENT department straight after the MRI examination to rule out magnet dislocation. In this context, transcutaneous ultrasound is a quick and reliable method for diagnosing magnet dislocation and treatment control [12].

Nowadays, the problem of magnet dislocation seems to have been overcome thanks to next-generation CI devices containing diametrical magnets not requiring a compression bandage up to 3 T that are available from all major manufacturers. Nevertheless, as the life expectancy of CI devices is several decades, this complication will still persist in future years.

### Limitations

The results of the study are limited by the fact that due to small subgroups, not all factors affecting the internal magnet during MRI examination could be considered, i.e. the type and duration of MRI sequences performed that can potentially influence the risk of dislocation. Additionally, some minor complications like pain during or after MRI could have been underestimated due to the retrospective character of the study. Furthermore, the dislocation rates of only three device types could be elaborated, as an insufficient number of scans were performed for the other devices. In this context, further preferably multicenter prospective studies should aim at a comparison with devices from Advanced Bionics® that also contain a removable axial magnet. In addition, the fact that the compression bandage was applied in different ways and by various physicians has a limiting effect on the results of the study. Apart from that, the large number of patients included and the direct comparison of dislocation rates for three different CI device types is a strength of the study.

## Conclusion

When MRI is performed at 1.5 T in CI with the internal magnet in place, complications occur in around 22%. The risk of magnet dislocation depends on the CI device type and ranges from 0 to 29.6%. Implants with a diametrical magnet showed no dislocations and can be regarded as potentially MRI-safe. On the contrary, in CI with axial magnets, the CI500 bears a high risk of magnet dislocation. Therefore, apart from a strict indication for MRI and adherence to the safety protocols, regular post-MRI follow-up examination of the respective implant is recommended to rule out magnet dislocation.

## Data Availability

The datasets used and analysed during the current study are available from the corresponding author on reasonable request.

## Abbreviations

CI: Cochlear implant
MRI: Magnetic resonance imaging
T: Tesla
